# “Turn-Off” Fluorescent Sensor for Pamidronate Disodium and Zoledronic Acid Based on Newly Synthesized Carbon Dots from Black Tea

**DOI:** 10.1155/2018/3631249

**Published:** 2018-03-13

**Authors:** Peng Li, Yongcheng Hu

**Affiliations:** ^1^The Graduate School, Tianjin Medical University, 22 Qixiangtai Road, Heping District, Tianjin 300071, China; ^2^Department of Orthopedic Oncology, Tianjin Hospital, 406 Jiefang South Road, Tianjin 300210, China

## Abstract

As common bisphosphonates drugs, pamidronate disodium and zoledronic acid have been widely investigated for bone metastases. In this paper, a new “turn-off” model based on carbon dots (CDs) from black tea was established to analyze the two kinds of bisphosphonate drugs, pamidronate disodium and zoledronic acid. Through the new sensor, both of drugs can be quantitative, respectively, with the limit of detection of 5 × 10^−9^ mol·L^−1^ and 6 × 10^−9^ mol·L^−1^. In addition, the fluorescence of newly prepared CDs can be quenched by two drugs with various degrees via photoinduced electron transfer, which can be perfectly used to distinguish them. Most importantly, this turn-off method has been employed to analyze the two drugs under the influence of foreign interference factors. This method provides a new view and guidance for the rapid analysis and recognition of drugs for bone metastases *in vitro* and *in vivo*.

## 1. Introduction

Due to their excellent photo stability, high luminous intensity, wide range of excitation spectrum, and narrow emission spectrum, carbon dots (CDs) have been applied as fluorescent sensor in many fields, such as biochemistry, food science, and medical analysis [1–5]. Recently, a kind of food-based carbon dots have attracted more and more attention because of its good biocompatibility, low toxicity, and easy preparation [6–8]. In the past, many reports of fluorescence detecting mode “turn-off” based on CDs have already been reported, such as the turn-off fluorescent sensor based on nitrogen-doped carbon dots applied to detect Hg^2+^ ions by Zhang and Chen [9] and the turn-off fluorescence sensor for the detection of ferric ion in water using green synthesized N-doped carbon dots [10].

As a significant kind of drugs, bisphosphonates play an important role in the treatment of metabolic bone diseases such as bone metasis and have been expanded widely to inhibit bone resorption and management of skeletal disorder including osteoporosis and malignant hypercalcemia [11, 12]. Meanwhile, as two representative drugs among the bisphosphonates, pamidronate disodium and zoledronic acid have been studied by more and more researchers [13, 14]. However, despite their frequent use, the rapid analytical method of them whether *in vitro* or *in vivo* has largely remained very few, and the traditional chromatographic methods were mostly time consuming and expensive and, what's more, have complicated pretreatments [15–17]. On the other hand, known as a fast, sensitive, and stable analytical technology, the fluorescent sensor based on quantum dots, especially carbon dots, have a good application prospect in the drug analysis [18, 19].

In this paper, a new turn-off sensor based on carbon dots from black tea was established to quantitatively characterize the two kinds of bisphosphonate drugs, pamidronate disodium and zoledronic acid. The morphology and chemical composition, stability, and fluorescence properties of the newly prepared CDs were analyzed in details. Through using the different binding ability between CDs and different drugs, this turn-off sensor can be successfully used to quantitatively analyze pamidronate disodium and zoledronic acid no matter under the influence of foreign interference factors or in the human plasma. Most importantly, the fluorescent sensor has been successfully used for the distinction of the two bisphosphonate drugs. This method illustrates its promising opportunities in the studies of other drugs *in vivo* or *in vitro* using the fluorescent sensor based on CDs.

## 2. Experimental

### 2.1. Experimental Materials and Reagents

The black tea was bought from Minghuang Natural Food Development Co., Ltd. Tris (hydroxymethyl) amino methane, HCl, KCl, NaCl, MgCl_2_, ZnCl_2_, CuCl_2_, CaCl_2_, and FeCl_3_ were purchased from Sinopharm Chemical Reagent Co., Ltd. CDs from the black tea via the hydrothermal route were synthesized in our laboratory. Deionized distilled water prepared from a Mole water purification system was used.

### 2.2. Apparatus and Procedures

UV-Vis absorption spectra were examined by using a U-3900 UV-Vis spectrometer (Hitachi ). The fluorescence spectra were acquired by an FL-7000 luminescence spectrometer (Hitachi ). High-resolution transmission electron microscopy (HRTEM) was examined by using a Tecnai G2 F30 S-Twin microscope (Philips-FEI ). Samples containing different concentrations of drugs and CDs were made up to 1 mL in 50 mmol·L^−1^ Tris-HCl (pH = 6.0). The concentration of CDs was set at 8.0 × 10^−5^ g·mL^−1^. The emission spectrum of the solution was then measured 5 min later. All optical measurements were performed at room temperature under ambient conditions, and the excitation wavelength (*λ*_ex_) was 360 nm.

### 2.3. Preparation of CDs from the Black Tea

The CDs were synthesized by using a facile, green, and low-cost hydrothermal treatment based on the black tea as the carbon source as follows: 0.5 g the black tea and 40 mL water were added into 50 mL reaction kettle and then heated at 180°C for 3 h. The obtained CDs were centrifuged for 15 min at a rotating speed of 5000 r·min^−1^ and then dialyzed in a dialysis bag (molecular weight cut off = 3500) for 12 h; finally, the product were freeze dried for further analysis.

### 2.4. Quantum Yield Measurement

The quantum yields (QYs) of the CDs were measured by comparing the integrated fluorescence intensity (320 nm excitation) and the absorbance value (320 nm) of CD and quinine sulfate was used as a reference. QY was calculated as follows:

(1) $$\begin{array}{c} Q_{\text{X}}=Q_{\text{ST}}\cdot \left(\frac{I_{\text{X}}}{I_{\text{ST}}}\right)\left(\frac{A_{\text{ST}}}{A_{\text{X}}}\right)\left(\frac{\eta_{\text{X}}^{2}}{\eta_{\text{ST}}^{2}}\right), \end{array}$$

where *I* is the integrated fluorescence intensity, *A* represents the absorbance value, and *η* means the refractive index of the solvent (both 1.33). The subscripts “X” and “ST” were CDs and quinine sulfate, respectively. The QY_ST_ was 54% while quinidine sulfate was in 0.1 mol·L^−1^ H_2_SO_4_. In order to minimize the absorption effect, the absorbance value was set as 0.1 [9].

## 3. Results and Discussion

### 3.1. Fluorescence Properties and Characterization of CDs

**Figure 1:**
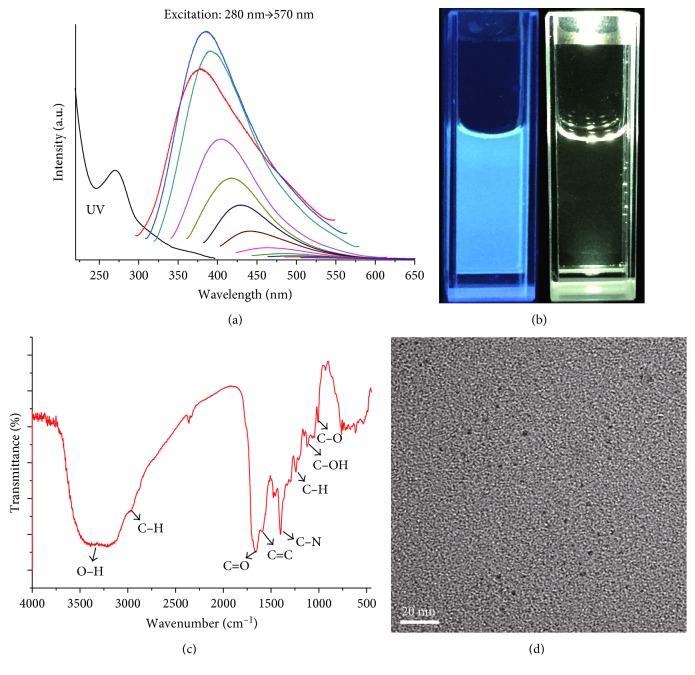
(a) UV-Vis spectrum of CDs (black line) and excitation-dependent of the CD fluorescence in water at room temperature. (b) The photograph of the CD solution under UV light of 365?nm (left) and under visible light (right). (c) FTIR spectra of CDs and (d) HRTEM images of CDs.

As shown in Figure 1(a), it can be seen that the absorbance band of CDs was centered at around 270 nm in the UV spectrum which was from the *n*-*π*^∗^ transition. The CDs emitted strong blue light under UV irradiation of 365 nm (Figure 1(b)), and while the *λ*_ex_ increased from 280 to 570 nm, the emission spectra of the CDs displayed a typical excitation wavelength-dependent characteristic, which had been suggested to be a result of varied fluorescence characteristics of CDs with various sizes or emissive sites existed on the surfaces of the CDs [2]. Chemical and structural information about the CDs using the FTIR spectroscopy was shown in Figure 1(c); the peak around 3214 cm^−1^ indicated the existence of −OH; the peaks around 2940 and 1240 cm^−1^ were assigned to the C−H; the peaks at 1660 cm^−1^ and 1620 cm^−1^ were attributed to the stretching vibration of C=O and C=C, respectively; the peaks at 1401 cm^−1^ were C−N stretching vibration modes; and the peaks at 1123 and 1007 cm^−1^ indicated C−OH and C−O, respectively.  Figure 1(d) shows the high-resolution TEM (HRTEM) image of the CDs which reveals that the CDs had a size distribution around 2.2 nm. According to (1), the QY of the CDs based on the black tea was calculated to be 10.23% with quinine sulfate as a reference.

**Figure 2:**
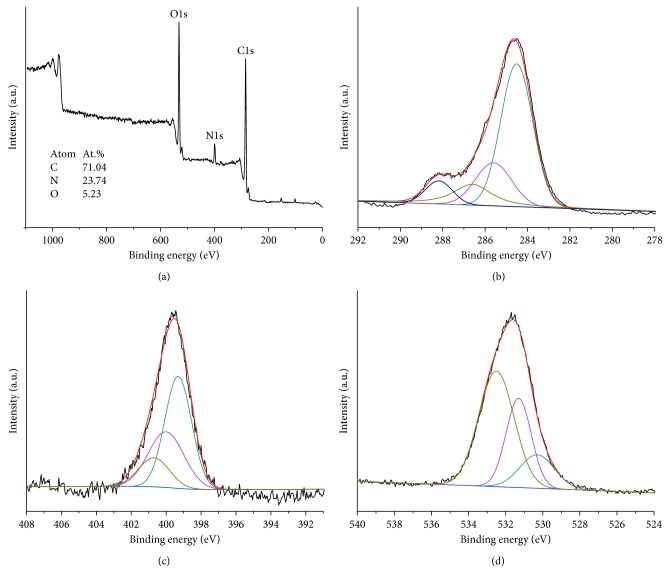
(a) XPS survey spectrum of CDs. (b) C1s, (c) O1s, and (d) N1s high-resolution XPS spectra of the CQDs.

In order to further study the structure of CDs, the XPS analysis was conducted.  Figure 2(a) shows the XPS spectrum of the CDs, and the three predominant peaks of C1s, N1s, and O1s at 284.61, 399.49, and 531.91 eV were corresponded to the binding energies. As can be seen from the result that the as-prepared CDs mainly contained C, O, and N, the contents of the three elements were calculated to be 73.8% (C), 21.7% (O), and 4.5% (N). The C1s spectrum (Figure 2(b)) can be deconvoluted into four peaks at around 284.6, 285.6, 286.6, and 287.9 eV, which were attributed to C–C, C–N, C–O, and C=O groups, respectively [9]. In addition, the N1s spectrum (Figure 2(c)) can be deconvoluted into three peaks at 399.3, 400.1, and 400.7 eV which can be assigned to N−(C)_3_, N−H, and C–N–H groups, respectively. From the O1s spectrum shown in Figure 2(d), the three fitted peaks at 530.3, 531.3, and 532.7 eV were ascribed to C−OH/C−O−C, adsorbed oxygen, and −C=O groups, respectively. The result from XPS showed that the surface of the CDs from the black tea was functionalized by multiple oxygenated and nitrous groups which may lead the interactions with different drug molecules.

### 3.2. Effective Quenching of Fluorescence of CDs by Pamidronate Disodium and Zoledronic Acid: Turn-Off Process

**Figure 3:**
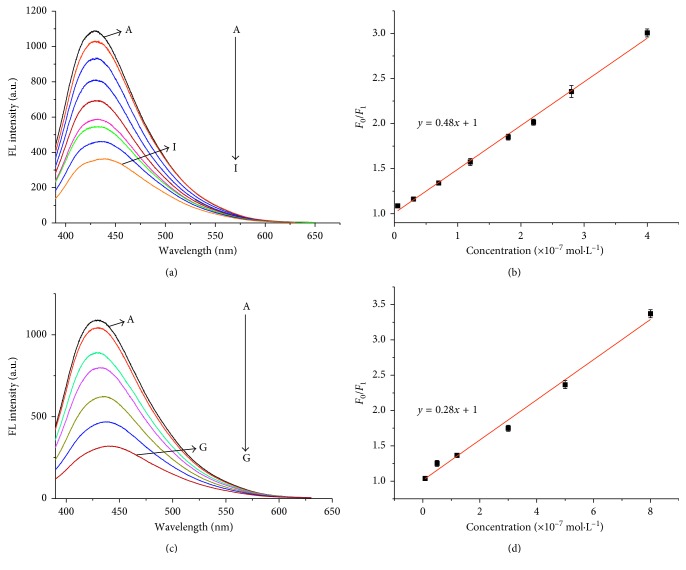
Fluorescence quenching behavior of CDs after the addition of various concentrations (5 × 10^−9^ mol·L^−1^ to 4 × 10^−7^ mol·L^−1^) of pamidronate disodium (A-I (a)) and the addition of various concentrations (6 × 10^−9^ mol·L^−1^ to 8 × 10^−7^ mol·L^−1^) of zoledronic acid (A-G (c)). The linear plot of relative fluorescence intensity (*F*_0_/*F*_1_) of CDs as a function of pamidronate disodium (5 × 10^−9^ mol·L^−1^ to 4 × 10^−7^ mol·L^−1^ (b) and zoledronic acid (6 × 10^−9^ mol·L^−1^ to 8 × 10^−7^ mol·L^−1^ (d)). Every data point was the mean of three measurements. The error bars were the standard deviation.

**Figure 4:**
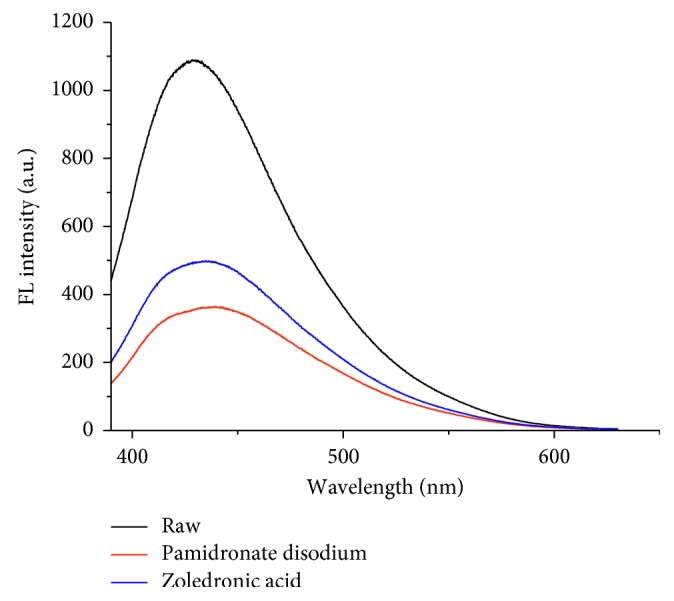
Fluorescence turn-off behavior of CDs in the presence of the same concentration of pamidronate disodium or zoledronic acid (4.0 × 10^−7^ mol·L^−1^).

**Scheme 1:**
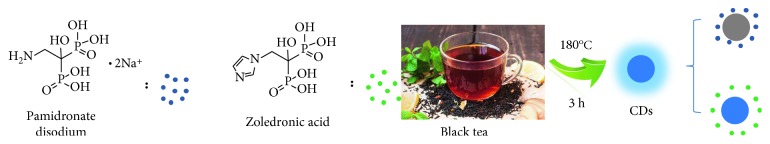
The chemical structure of pamidronate disodium or zoledronic acid and the illustration of turn-off fluorescent sensor for pamidronate disodium and zoledronic acid based on carbon dots from black tea.

**Figure 5:**
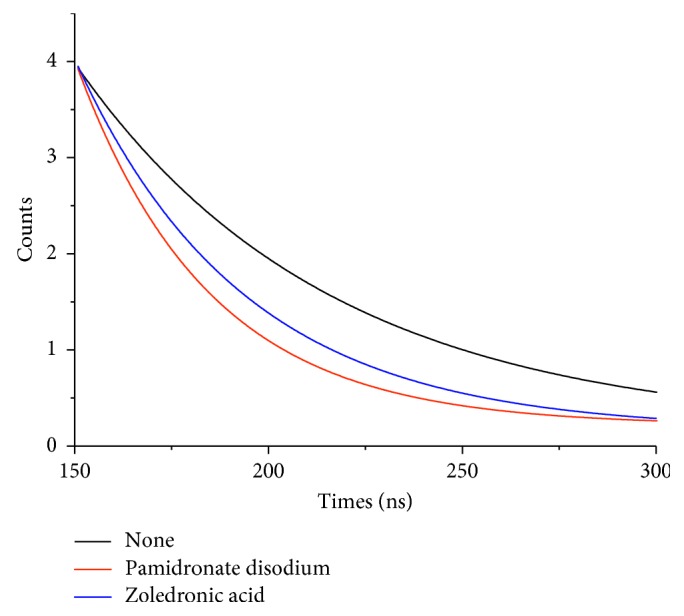
The lifetime of CDs after adding different bisphosphonates.

As shown in Figure 3(a), the increased addition of pamidronate disodium led to the gradual decreased fluorescence of CDs. And the relationship between the concentration of pamidronate disodium and the fluorescence intensities of CDs follows the “Stern-Volmer” equation:

(2) $$\begin{array}{c} \frac{F_{0}}{F_{1}}=1+K_{\text{sv}}\left[\text{M}\right], \end{array}$$

where *F*_0_ and *F*_1_ were the fluorescence intensities of CDs in the absence and presence of the drugs, respectively; [M] was the concentration of pamidronate disodium, and the quenching constant *K*_sv_ defines the quenching efficiency of pamidronate disodium. The equation shows that *F*_0_/*F*_1_ was directly proportional to the concentration of pamidronate disodium. As shown in the Stern-Volmer plot in the inset of Figure 1, the *K*_sv_ of pamidronate disodium to the fluorescence of CDs was 4.8 × 10^6^ L·mol^−1^, with linear range from 5 × 10^−9^ mol·L^−1^ to 4 × 10^−7^ mol·L^−1^ and a correlation coefficient *R* = 0.996. On the other hand, as shown in Figure 3(c), when the concentration of zoledronic acid was increasing from 6 × 10^−9^ mol·L^−1^ to 8 × 10^−5^ mol·L^−1^, the fluorescent intensity of the CDs at around 430 nm also decreases gradually, which indicating that zoledronic acid can effectively quench the fluorescence of CDs. Meanwhile, through the Stern-Volmer equation (Figure 3(d)), the *K*_sv_ of zoledronic acid to the fluorescence of CDs was 2.8 × 10^6^ L·mol^−1^ with the linear range from 6 × 10^−9^ mol·L^−1^ to 8 × 10^−7^ mol·L^−1^ and a correlation coefficient *R* = 0.991. The detect limit of pamidronate disodium and zoledronic acid was, respectively, 5 × 10^−9^ mol·L^−1^ and 6 × 10^−9^ mol·L^−1^. Obviously, pamidronate disodium exhibits better quenching ability to the fluorescence of CDs than zoledronic acid with the value of *K*_sv_ almost twice higher than that of zoledronic acid and seen in Figure 4, the addition of pamidronate disodium shows a significantly weaker fluorescence quenching phenomenon than the same concentration of zoledronic acid, which may be caused by the different intermolecular binding capacities. As shown in Scheme 1, compared with the imidazolyl group contained in zoledronic acid, the amino group contained in pamidronate disodium has the smaller space obstruction and a better affinity to the polycarboxyl group modified on the surface of the CDs, leading to much stronger quenching phenomenon to the fluorescence via photoinduced electron transfer and exhibiting excellent selectivity in detecting the two drugs with similar structures. To explain the sensing mechanism in depth, lifetimes of CDs before and after adding bisphosphonates were investigated and the result was shown in Figure 5. The lifetime of CDs after adding different bisphosphonates was reduced by different degrees which can act as a proof for the photoinduced electron transfer mechanism. And what's more, the reduction fluorescence lifetime of CDs caused by pamidronate disodium was more than that caused by zoledronic acid. This also demonstrated the different quenching capabilities of bisphosphonates towards the carbon dots.

### 3.3. Selectivity of the Sensor for Pamidronate Disodium and Zoledronic Acid Detection

**Figure 6:**
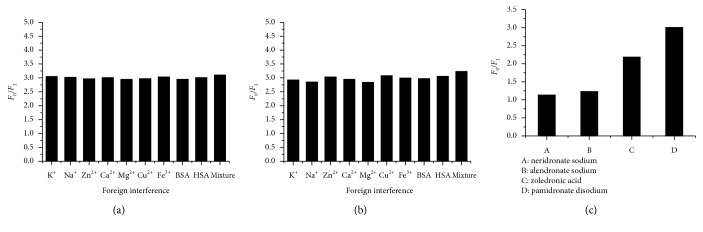
The influence of naturally abundant foreign metal ions and biomolecules (1.0 × 10^−5^ mol·L^−1^) for the turn-off sensor ((a) pamidronate disodium: 4 × 10^−7^ mol·L^−1^ and (b) zoledronic acid: 8 × 10^−7^ mol·L^−1^) and the comparison of different quenching abilities towards CDs of different bisphosphonates at the concentration of 4 × 10^−7^ mol·L^−1^ (c).

As to evaluate the selectivity of the CDs sensor, 10 different biologically relevant metal ions including Na^+^, K^+^, Mg^2+^, Ca^2+^, Fe^3+^, Zn^2+^, Cu^2+^, Bovine serum albumin (BSA), human serum albumin (HSA), and the mixture of all the above were selected as foreign interference with a concentration at 1 × 10^−5^ mol·L^−1^. The comparison is shown in Figure 6 which can indicate that the interference effect of all the 10 interferences were slight. In addition, even in the spiked samples of the human plasma and urine, a recovery of 101.3% and 99.4% for pamidronate disodium (4 × 10^−7^ mol·L^−1^) and a recovery of 102.1% and 100.8% for zoledronic acid (8 × 10^−7^ mol·L^−1^) were obtained. What's more, other kinds of bisphosphonates such as neridronate sodium and alendronate sodium also failed to have a significant impact on the fluorescence of CDs. It was obvious that a special effect appeared between CDs and pamidronate disodium or zoledronic acid, so the as-prepared CDs had a high selectivity for pamidronate disodium and zoledronic acid analysis.

## 4. Conclusion

In this study, a new turn-off model based on newly prepared carbon dots (CDs) from the black tea was established to analyze pamidronate disodium and zoledronic acid. By using the new sensor, both of drugs can be quantitative, respectively, with high sensitivity and stability. Meanwhile, the fluorescence of the CDs can be quenched by two drugs with various degrees via photoinduced electron transfer, which provides the sensor a high selectivity to the two structurally similar drugs. What's more, this turn-off method shows good stability and has been employed to analyze the two drugs under the influence of common foreign interference factors which indicating its good application prospects for drugs analysis *in vitro* and *in vivo*.
